# Regional differences in mental health stigma—Analysis of nationally representative data from the Health Survey for England, 2014

**DOI:** 10.1371/journal.pone.0210834

**Published:** 2019-01-22

**Authors:** Vishal Bhavsar, Peter Schofield, Jayati Das-Munshi, Claire Henderson

**Affiliations:** 1 Section of Women’s Mental Health, Health Services and Population Research Department, King’s College London Institute of Psychiatry, Psychology and Neuroscience, London, United Kingdom; 2 Department of Population Health Sciences, School of Population Health & Environmental Sciences, King’s College London, London, United Kingdom; 3 Heath Services and Population Research Department, King’s College London Institute of Psychiatry, Psychology and Neuroscience, London, United Kingdom; 4 South London & Maudsley NHS Foundation Trust, London, United Kingdom; St. George’s University of London, UNITED KINGDOM

## Abstract

**Background:**

Mental health stigma persists despite coordinated and widely-evaluated interventions. Socioeconomic, structural, and regional context may be important in shaping attitudes to mental illness, and response to stigma interventions. Regional differences in attitudes towards mental illness could be relevant for intervention, but have not been systematically explored. We evaluated regional variation in mental health stigma using nationally representative data from England, the Health Survey for England (HSE), from 2014.

**Methods:**

A previously derived scale for mental health-related attitudes with 2 factors (i. tolerance and support, ii. prejudice and exclusion), and overall attitudes, were outcomes. Weighted linear regressions estimated contribution of individual characteristics and region of residence to inter-individual variability in mental health-related attitudes.

**Results:**

London and southern regions tended to have more negative mental health-related attitudes. These differences were not fully or consistently explained by individual sociodemographic characteristics, or personal familiarity with mental illness.

**Conclusions:**

Stigma policies could require refinements based on geographic setting. Regions may be in particular need of stigma interventions, or be more resistant to them. Regional differences might be related to media coverage of mental illness, funding differences, service availability, or accessibility of educational opportunities. Greater geographic detail is necessary to examine reasons for regional variation in stigmatizing attitudes towards people with mental illness, for example through multilevel analysis.

## Introduction

Improving attitudes to mental illness among the general public could help to address unmet mental health need, improve the accessibility and take-up of mental healthcare and improve outcomes of mental illness[1]. Although reducing mental health stigma is a priority for mental health services and policy makers, there is evidence that stigmatizing attitudes towards mental illness persist[2], and that enduring improvements to attitudes in response to anti-stigma campaigns have been limited.

Current theoretical explanations for negative attitudes towards people with mental illness in the general population emphasise differences in individual characteristics, and a person’s familiarity with mental illness. In line with this, evidence suggest that attitudes towards mental illness are more positive in those suffering personally from mental illness, or who know someone with mental illness, such as a friend or family member[3, 4]. Men report more negative attitudes towards people with mental illness compared to women, in most studies[3, 5–7]. Surveys have also reported differences in mental health stigma between ethnic groups[3], possibly indicating that attitudes towards mental illness might also be shaped by cultural background. There is also a reported association between mental health-related attitudes and socioeconomic indices, with possibly more negative attitudes reported in those of lower socioeconomic position, or exposed to greater socioeconomic deprivation and disadvantage[8]. However, some studies do not suggest this association, particularly those from poorer countries[9, 10].

Attitudes towards mental illness might also be influenced by characteristics of the communities or groups in which people live, work and study. Such characteristics include the distribution of wealth, services (including health services), social support, and educational opportunities, which typically vary between geographic areas[11]. Geographic areas with greater social disorder and fear of crime[12, 13] could engender more negative attitudes towards people with mental illness by heightening underlying beliefs that people with mental illness commit more crimes; this could represent a mechanism by which geographic areas influence negative attitudes towards people with mental illness. Alternatively, an individual’s expression of stigmatizing attitudes towards people with mental illness could be a reflection of the local prevalence of and familiarity with people with mental illness in their area, and local news reporting of incidents of crime and violence perpetrated by people with mental illness[14]. Such incidents might also be more common, or create more publicity, where mental health care receives less state funding, or where quality of care is perceived as worse[15]. In particular, instances of crimes committed by people with mental illness could have negative influence on attitudes in areas closest to the locations where such incidents occurred, either due to greater news reporting or to greater affinity residents might feel with events occurring in their own region, as opposed to nationally.

Finding regional differences in mental health-related attitudes would suggest greater emphasis on understanding the social and environmental context within which anti-stigma campaigns are delivered. Evidence on the possible role of structural and ecological characteristics, such as deprivation, cohesion, social fragmentation, neighbourhood social disorder (possibly reflected in levels of local crime, anti-social behaviour, and fear of crime) and the physical environment, in shaping individual mental health stigma could allow development of targeted group-level interventions. Moreover, the pervasiveness of mental health stigma despite campaigns in some settings could be partly explained by social environment, and therefore be assisted by adapting campaigns to the local geographic context. Geographic variation could also indicate clues as to structural factors that underlie stigmatizing attitudes towards mental illness in the general population.

Therefore, in this study we evaluated regional differences in mental health related attitudes in England using representative data from an English household survey, aiming to estimate differences in mental health-related attitudes between regions of England, and the contribution of individual characteristics to these differences.

## Materials and methods

### Sampling

The Health Surveys for England are nationally representative household surveys of the private residential population, collecting information on health, sociodemographic characteristics and attitudes[16]. The annual surveys contain a core set of questions asked every year, together with year-specific items assessing different themes. In the 2014 survey, items assessing attitudes to mental illness were collected and are described below (see Measurements). Information on attitudes was not available in the other years of the survey, therefore we only analysed data from respondents to the HSE 2014, aged 16 and over. A two-stage stratified random sampling process was used, first sampling postcode sectors, and then households from each sampled postcode sector, with the small-user postcode address file (PAF) as the sampling frame[16, 17]. Information on familiarity with mental illness(described below, under *Covariates*) was derived from nurse visit data, and data on other covariates obtained by household interview. We therefore employed the nurse interview weighting, in line with previous analysis of HSE 2014 data[18]. Weights corrected the distribution of household members to match population estimates for sex/age groups and region, and accounted for non-response within households, and for non-response to the nursing interview.

### Measurements

#### Regions

Each respondent was classified into English standard government office regions using a nine-category nominal variable, for those residing in London, South East England, South West England, the East of England, the East Midlands, the West Midlands, North East England, North West England, and Yorkshire and Humber. This regional classification has been used in the previous HSE sweeps[16], and in other nationally representative epidemiological studies on mental health, such as the Adult Psychiatric Morbidity Surveys[19].

#### Mental health-related attitudes

Mental health-related attitudes were assessed by the Scale for Community Attitudes toward the Mentally Ill (abbreviated to CAMI in this report), developed in 1981 to measure community attitudes towards people with mental illness[20] by ascertaining agreement with a series of statements about mental illness, by Likert scale. For HSE 2014, a 12-item version of the scale (CAMI–12) was used at stage 1 of sampling, and comprised a subset of the original statements, selected to measure levels of mental health-related stigma and tolerance. Items refer to attitudes on social exclusion, benevolence, tolerance, and support for people affected by mental illness, each rated from 1(strong disagreement) to 5(strong agreement). These statements included: "one of the main causes of mental illness is a lack of self-discipline and willpower", "there is something about people with mental illness that makes it easy to tell them apart from other people", "I would not want to live next door to someone who has been mentally ill", and "people with mental illness should not be given any responsibility". This approach was first used in the evaluation of the Time to Change campaign[21]. The CAMI questionnaire was administered to HSE respondents as part of the self-completion questionnaire during the interview visit. To derive overall measures of stigma for analysis, exploratory factor analysis was performed on data for the 12 CAMI items in HSE 2014, producing two factors, reflecting themes of i. prejudice and exclusion, and ii. tolerance and support for community care. The two factors were internally reliable, with Cronbach’s alpha greater than 0.6 for both factors[17]. The two factor solution was consistent with previous analysis of the CAMI[22]. All items were scored so that increasing factor scores reflected more positive attitudes on that characteristic that is, greater tolerance and support, or lower prejudice and exclusion. Factor scores were z-standardized, to have a mean of 0 and a standard deviation of 1. The two factors were combined to provide an overall mental health stigma score, by taking the mean of the two scores.

#### Covariates

Age was in ten-year groups from 16. Ethnicity was classified into white, black, Asian, mixed, and other ethnic group. Highest educational attainment was categorised into no qualifications, qualifications below degree level, and degree level or equivalent. For description, income data was grouped into categories for weekly incomes of 0-£232.99, £233–368.99, £369–531.99, £532–851.99, and £852 and above. Residential neighbourhood deprivation was measured by linking the postcode of the respondent to national data on neighbourhood deprivation score from the Office for National Statistics, classifying each respondent into quintiles of neighbourhood deprivation. Extent of familiarity with mental illness was assessed by an item asking “who is the person closest to you who has or has had some kind of mental illness?”, with responses reflecting a. having mental illness oneself, b. someone else with mental illness, c. a partner with mental illness, d. another family/friend with mental illness, e. an acquaintance, work colleague, or other acquaintance with mental illness, and finally f. not knowing anyone with mental illness. For the present study, these categories were aggregated to form a three-level categorical variable for familiarity reflecting either a. having mental illness yourself, b. knowing someone with mental illness, or c. not knowing anyone with mental illness. Urban/rural status of the household was classified according to the 2011 UK census, classified at the “Super Output Area” geographic level[23]. To assess linearity, models with linear and non-linear terms for age and income were compared using Wald tests, suggesting linear handling of income, and indicator variables for each ten-year category of age, in modelling.

### Analysis

Data was analysed using STATA 14[24]. All analyses incorporated survey weights with robust standard errors, to account for non-response and clustering of responses within households. Distributions of mean scores on tolerance and support, prejudice and exclusion, and overall mental health-related attitudes were inspected by study covariates (Table 1). Associations between each covariate and attitudes were examined using linear regressions, unadjusted and adjusted for age and gender (Table 2). We then carried out analysis of region level differences in attitudes(Table 3)—standardized mean differences in stigma predicted by region were estimated in multivariable models. Multilevel analysis was not used, owing to the small number of geographic units. Adjustments in multivariable models were made, in turn, for: i. basic demographic variables (which were age, gender, and ethnicity) ii. socioeconomic characteristics(education and income), and finally iii. extent of familiarity with mental illness. All variables were modelled as indicator categorical variables with the exception of income, which was modelled as continuous. Neighbourhood deprivation and urban-rural status were not included in models, as they were geographically clustered and therefore violated the random sampling assumptions of fixed effects regression models. Full model estimates from final models are presented in Table 4. Based on the final analytic sample, distribution of mean scores on mental health-related attitudes by each covariate were inspected (Table 5).

**Table 1:** Description of study population in terms of mental health-related attitudes, based on responses to the CAMI, from the overall sample (N = 1080). Footnotes describe numbers of participants for each estimate.

	Overall frequency (weighted %)	Tolerance and support^a^		Prejudice and exclusion^a^		Overall CAMI^a^	
		Mean	(95% CI)	Mean	(95% CI)	Mean	(95% CI)
**Region**							
North East	964(4.88)	0.07	(-0.04,0.18)	0.11	(0.00,0.22)	0.09	(-0.01,0.19)
North West	1337(13.37)	-0.07	(-0.20,0.07)	-0.09	(-0.20,0.03)	-0.07	(-0.18,0.03)
Yorkshire and Humber	891(9.82)	0.02	(-0.09,0.13)	0.06	(-0.05,0.16)	0.05	(-0.04,0.14)
East Midlands	893(8.59)	0.01	(-0.11,0.12)	0.05	(-0.06,0.17)	0.03	(-0.07,0.13)
West Midlands	966(10.47)	0.01	(-0.11,0.13)	0.02	(-0.09,0.13)	0.01	(-0.09,0.12)
East of England	1232(11.22)	0.06	(-0.02,0.13)	0.10	(0.03,0.18)	0.09	(0.03,0.15)
London	1172(15.27)	-0.15	(-0.27,-0.03)	-0.07	(-0.21,0.08)	-0.11	(-0.22,0.01)
South East	1635(16.34)	0.01	(-0.08,0.09)	0.04	(-0.04,0.12)	0.02	(-0.05,0.09)
South West	990(10.04)	-0.08	(-0.17,0.02)^b^	-0.08	(-0.21,0.06)^c^	-0.08	(-0.18,0.03)^d^
**Age**							
16–24	780(14.22)	-0.37	(-0.50,-0.24)	0.01	(-0.11,0.12)	-0.18	(-0.29,-0.07)
25–34	1128(16.85)	-0.14	(-0.23,-0.05)	0.01	(-0.10,0.11)	-0.06	(-0.14,0.02)
35–44	1410(16.61)	0.01	(-0.08,0.10)	0.05	(-0.02,0.13)	0.03	(-0.04,0.11)
45–54	1481(17.54)	0.04	(-0.03,0.12)	0.19	(0.11,0.26)	0.12	(0.05,0.19)
55–64	1209(14.02)	0.15	(0.08,0.22)	0.09	(0.01,0.16)	0.12	(0.06,0.18)
65–74	1190(11.53)	0.10	(0.03,0.17)	-0.13	(-0.22,-0.03)	-0.01	(-0.08,0.06)
75+	879(9.23)	0.09	(0.00,0.18)^e^	-0.43	(-0.52,-0.34)^f^	-0.17	(-0.24,-0.09)^g^
**Gender**							
Male	4624(49.34)	-0.11	(-0.16,-0.06)	-0.13	(-0.18,-0.08)	-0.12	(-0.16,-0.08)
Female	5456(50.66)	0.05	(0.01,0.10)^h^	0.13	(0.09,0.18)^i^	0.10^j^	(0.06,0.14)^j^
**Education**							
Degree or equivalent	2037(26.46)	0.17	(0.10,0.23)	0.26	(0.19,0.32)	0.21	(0.16,0.27)
Below degree	4133(52.37)	-0.06	(-0.11,-0.02)	0.02	(-0.03,0.07)	-0.02	(-0.06,0.02)
None	1868(21.17)	-0.21	(-0.31,-0.11)^k^	-0.44	(-0.52,-0.36)^l^	-0.32	(-0.39,-0.26)^m^
**Ethnicity**							
White	8709(84.85)	0.03	(-0.01,0.06)	0.07	(0.04,0.11)	0.05	(0.02,0.08)
Black	275(2.86)	-0.38	(-0.62,-0.13)	-0.49	(-0.76,-0.22)	-0.42	(-0.65,-0.20)
Asian	730(8.71)	-0.49	(-0.70,-0.28)	-0.63	(-0.86,-0.41)	-0.56	(-0.71,-0.40)
Mixed	230(2.43)	-0.57	(-0.88,-0.25)	-0.17	(-0.50,0.16)	-0.34	(-0.64,-0.03)
Other	97(1.13)	-0.35	(-0.81,0.11)^n^	-0.44	(-0.79,-0.10)^o^	-0.39	(-0.77,-0.01)^p^
**Income**^ac^							
0.00–232.99	1546(16.75)	-0.13	(-0.23,-0.02)	-0.16	(-0.26,-0.05)	-0.13	(-0.21,-0.05)
233.00–368.99	1337(14.21)	-0.11	(-0.21,-0.01)	-0.17	(-0.25,-0.08)	-0.13	(-0.21,-0.05)
369.00–531.99	1660(10.82)	-0.03	(-0.11,0.05)	-0.04	(-0.12,0.04)	-0.03	(-0.11,0.04)
532.00–851.99	1797(20.82)	0.05	(-0.03,0.12)	0.16	(0.08,0.23)	0.10	(0.04,0.17)
852.00 and above	1874(24.81)	0.14	(0.07,0.21)^q^	0.23	(0.16,0.29)^r^	0.18	(0.130.24)^s^
**Neighbourhood deprivation**							
Least	2235(21.55)	0.10	(0.04,0.16)	0.16	(0.09,0.23)	0.13	(0.07,0.19)
	1945(18.75)	0.01	(-0.07,0.08)	0.03	(-0.04,0.11)	0.02	(-0.04,0.09)
	1905(18.90)	0.03	(-0.04,0.10)	0.04	(-0.03,0.11)	0.04	(-0.02,0.10)
	2002(20.63)	-0.10	(-0.19,-0.02)	-0.08	(-0.18,0.02)	-0.09	(-0.17,-0.01)
Most	1993(20.17)	-0.20	(-0.32,-0.09)^t^	-0.17	(-0.26,-0.07)^u^	-0.18	(-0.26,-0.09)^v^
**Urban-rural**							
City	8125(81.92)	-0.05	(-0.09,-0.01)	-0.02	(-0.06,0.03)	-0.03	(-0.07,0.00)
Town or fringe	987(8.96)	0.03	(-0.04,0.11)	0.08	(-0.01,0.18)	0.07	(0.00,0.14)
Village or hamlet	968(9.12)	0.13	(0.04,0.23)^w^	0.15	(0.06,0.24)^x^	0.14	(0.06,0.22)^y^
**Familiarity with mental illness**							
Yourself	234(3.83)	0.11	(-0.03,0.26)	0.20	(0.05,0.35)	0.16	(0.03,0.28)
Other	3404(62.54)	0.09	(0.05,0.13)	0.19	(0.15,0.23)	0.14	(0.11,0.18)
Don’t know anyone with mental illness	1843(33.62)	-0.28	(-0.35,-0.21)^z^	-0.40	(-0.46,-0.33)^aa^	-0.33	(-0.39,-0.28)^ab^

^a^. Two dimensions of mental health-related attitudes, tolerance and support, and prejudice and exclusion, were derived from factor analysis of responses to the CAMI, a structured tool assessing attitudes towards people with mental illness. Scores on the two dimensions were averaged to create a score for the overall CAMI. Higher scores indicate more positive attitudes

^b^. Based on a total of 8368 records with complete data,

^c^. n = 8357

^d^. n = 8333

^e^. n = 7614

^f^. n = 7603

^g^. n = 7579

^h^. n = 8368

^i^. n = 8357

^j^. n = 8333

^k^. n = 7579

^l^. n = 7568

^m^. n = 7544

^n^. n = 8330

^o^. n = 8319

^p^. n = 8295

^q^. n = 6783

^r^. n = 6770

^s^. n = 6755

^t^. n = 8368

^u^. n = 8357

^v^. n = 8333

^w^. n = 8368

^x^. n = 8357

^y^. n = 8333

^z^. n = 5021

^aa^. n = 5010

^ab^.n = 4986.

^ac^. Income is described in units of British pounds for weekly salary; in regression models income was included as a continuous variable for annual salary in thousands, to aid interpretation of estimates.

**Table 2:** Unadjusted, and age- and gender-adjusted associations(standardized mean differences) between attitude scores and each covariate, weighted for survey design and non-response.

	Tolerance and support		Tolerance and support^a^		Prejudice and exclusion		Prejudice and exclusion^a^		Overall CAMI		Overall CAMI^a^	
**Age**												
16–24	Reference		Reference		Reference		Reference		Reference		Reference	
25–34	0.23	(0.07,0.39)	0.22	(0.06,0.39)	0.00	(-0.16,0.15)	-0.01	(-0.160.14)	0.12	(-0.01,0.25)	0.12	(-0.02,0.25)
35–44	0.38	(0.22,0.53)	0.37	(0.220.53)	0.05	(-0.09,0.18)	0.04	(-0.090.17)	0.21	(0.09,0.34)	0.21	(0.08,0.33)
45–54	0.41	(0.26,0.57)	0.41	(0.250.56)	0.18	(0.05,0.31)	0.17	(0.050.30)	0.30	(0.18,0.42)	0.30	(0.17,0.42)
55–64	0.52	(0.38,0.66)	0.51	(0.370.66)	0.08	(-0.06,0.22)	0.07	(-0.060.21)	0.30	(0.18,0.42)	0.29	(0.18,0.41)
65–74	0.47	(0.32,0.62)	0.46	(0.320.61)	-0.13	(-0.28,0.02)	-0.14	(-0.290.01)	0.17	(0.04,0.29)	0.16	(0.03,0.29)
75+	0.46	(0.30,0.61)	0.44	(0.290.60)	-0.43	(-0.58,-0.29)	-0.45	(-0.60–0.31)	0.01	(-0.11,0.14)	0.00	(-0.13,0.12)
**Gender**												
Male	Reference		Reference		Reference		Reference		Reference		Reference	
Female	0.16	(0.10,0.23)	0.16	(0.090.22)	0.26	(0.20,0.32)	0.27	(0.21,0.33)	0.22	(0.17,0.27)	0.22	(0.17,0.27)
**Education**												
Degree	Reference		Reference		Reference		Reference		Reference		Reference	
Below degree	-0.23	(-0.30,-0.15)	-0.23	(-0.31–0.16)	-0.24	(-0.31,-0.16)	-0.25	(-0.33,-0.18)	-0.23	(-0.29,-0.17)	-0.24	(-0.30,-0.18)
No qualification	-0.37	(-0.49,-0.26)	-0.53	(-0.66–0.40)	-0.70	(-0.79,-0.60)	-0.66	(-0.76,-0.56)	-0.53	(-0.62–0.45)	-0.59	(-0.68,-0.51)
**Ethnicity**												
White	Reference		Reference		Reference		Reference		Reference		Reference	
Black	-0.40	(-0.66,-0.15)	-0.40	(-0.65–0.15)	-0.56	(-0.83,-0.29)	-0.63	(-0.89,-0.37)	-0.48	(-0.70–0.25)	-0.51	(-0.73,-0.29)
Asian	-0.52	(-0.73,-0.31)	-0.44	(-0.65–0.23)	-0.71	(-0.93,-0.48)	-0.75	(-0.98,-0.52)	-0.61	(-0.77–0.45)	-0.59	(-0.76,-0.42)
Mixed	-0.60	(-0.91,-0.28)	-0.52	(-0.84–0.21)	-0.24	(-0.580.09)	-0.29	(-0.64,0.06)	-0.39	(-0.69–0.09)	-0.37	(-0.68,-0.06)
Other	-0.38	(-0.84,0.08)	-0.33	(-0.820.15)	-0.52	(-0.86–0.17)	-0.57	(-0.92,-0.22)	-0.44	(-0.82–0.06)	-0.44	(-0.83,-0.05)
**Urban-Rural**												
Urban	Reference											
Town	0.08	(-0.00,0.17)	0.05	(-0.03,0.13)	0.10	(0.00,0.21)	0.12	(0.02,0.22)	0.10	(0.02,0.18)	0.09	(0.02,0.17)
Village	0.18	(0.08,0.29)	0.14	(0.03,0.25)	0.17	(0.07,0.26)	0.16	(0.07,0.26)	0.17	(0.08,0.26)	0.15	(0.06,0.24)
**Neighbourhood deprivation**												
Least deprived	Reference		Reference		Reference		Reference		Reference		Reference	
	-0.09	(-0.18,0.00)	-0.10	(-0.190.00)	-0.13	(-0.23,-0.02)	-0.11	(-0.21,-0.01)	-0.11	(-0.19–0.02)	-0.10	(-0.19,-0.02)
	-0.07	(-0.17,0.03)	-0.07	(-0.160.03)	-0.12	(-0.22,-0.01)	-0.12	(-0.22,-0.01)	-0.09	(-0.18–0.01)	-0.09	(-0.18,-0.01)
	-0.20	(-0.30,-0.10)	-0.16	(-0.26–0.06)	-0.24	(-0.36,-0.11)	-0.25	(-0.37,-0.13)	-0.22	(-0.32–0.12)	-0.21	(-0.30,-0.11)
Most deprived	-0.30	(-0.43,-0.17)	-0.27	(-0.40–0.14)	-0.32	(-0.44,-0.20)	-0.35	(-0.47,-0.23)	-0.31	(-0.41–0.21)	-0.30	(-0.40,-0.20)
**Income**												
0.00–232.99	Reference		Reference		Reference		Reference		Reference		Reference	
233.00–368.99	0.01	(-0.13,0.16)	0.00	(-0.150.15)	-0.01	(-0.15,0.13)	0.00	(-0.14,0.14)	0.00	(-0.120.11)	0.00	(-0.12,0.11)
369.00–531.99	0.10	(-0.03,0.23)	0.07	(-0.070.20)	0.11	(-0.02,0.24)	0.13	(0.00,0.25)	0.10	(-0.010.20)	0.09	(-0.02,0.20)
532.00–851.99	0.17	(0.04,0.30)	0.18	(0.050.32)	0.31	(0.17,0.45)	0.31	(0.17,0.44)	0.24	(0.130.34)	0.24	(0.13,0.35)
852.00 and above	0.27	(0.14,0.40)	0.27	(0.130.40)	0.38	(0.26,0.50)	0.36	(0.24,0.48)	0.32	(0.220.42)	0.30	(0.20,0.41)
**Familiarity**												
Self	Reference		Reference		Reference		Reference		Reference		Reference	
Other	-0.02	(-0.18,0.13)	0.02	(-0.13,0.17)	0.00	(-0.16,0.15)	0.03	(-0.13,0.19)	-0.01	(-0.14,0.12)	0.03	(-0.11,0.16)
No one	-0.39	(-0.59,-0.23)	-0.36	(-0.53,-0.20)	-0.60	(-0.76,-0.44)	-0.51	(-0.67,-0.35)	-0.49	(-0.63,-0.35)	-0.43	(-0.57,-0.29)

^a^. Adjusted for age and gender

b. Weekly income in British pounds

**Table 3:** Partial- and fully adjusted estimates for the association between region and mental health-related attitudes, weighted for clustered sampling and non-response. Models are based on 4277 participants with complete data on modelled variables.

	Unadjusted		+age, gender, ethnicity		+education, income^b^		+familiarity with mental illness	
Tolerance and support	Mean difference (95% CI)		Mean difference (95% CI)		Mean difference (95% CI)		Mean difference (95% CI)	
North East	Reference		Reference		Reference		Reference^c^	
North West	-0.16	(-0.32,0.00)	-0.11	(-0.26,0.03)	-0.12	(-0.26,0.01)	-0.11	(-0.24,0.02)
Yorkshire and Humber	-0.06	(-0.21,0.10)	-0.04	(-0.19,0.11)	-0.06	(-0.20,0.08)	-0.04	(-0.18,0.09)
East Midlands	-0.11	(-0.25,0.04)	-0.09	(-0.24,0.05)	-0.11	(-0.25,0.03)	-0.09	(-0.22,0.05)
West Midlands	-0.06	(-0.22,0.11)	-0.05	(-0.21,0.11)	-0.06	(-0.21,0.09)	-0.05	(-0.20,0.10)
East of England	-0.04	(-0.17,0.09)	-0.02	(-0.14,0.10)	-0.03	(-0.15,0.08)	-0.02	(-0.13,0.09)
London	-0.23	(-0.39, -0.06)	-0.08	(-0.24,0.09)	-0.15	(-0.30,0.01)	-0.13	(-0.29,0.03)
South East	-0.12	(-0.26,0.02)	-0.11	(-0.24,0.02)	-0.15	(-0.27,-0.03)	-0.14	(-0.26,-0.02)
South West	-0.22	(-0.37,-0.07)	-0.21	(-0.35,-0.06)	-0.23	(-0.37,-0.09)	-0.23	(-0.37,-0.09)
**Prejudice and exclusion**								
North East	Reference		Reference		Reference		Reference^d^	
North West	-0.23	(-0.40,-0.07)	-0.13	(-0.29,0.02)	-0.14	(-0.29,0.00)	-0.13	(-0.27,0.01)
Yorkshire and Humber	-0.05	(-0.20,0.09)	-0.03	(-0.17,0.12)	-0.05	(-0.19,0.09)	-0.03	(-0.17,0.10)
East Midlands	-0.13	(-0.27,0.02)	-0.09	(-0.23,0.06)	-0.11	(-0.25,0.03)	-0.08	(-0.21,0.05)
West Midlands	-0.11	(-0.28,0.06)	-0.07	(-0.23,0.09)	-0.09	(-0.23,0.06)	-0.08	(-0.22,0.06)
East of England	-0.05	(-0.17,0.08)	-0.02	(-0.14, 0.10)	-0.04	(-0.16,0.07)	-0.03	(-0.14,0.08)
London	-0.19	(-0.36,-0.02)	-0.02	(-0.17,0.12)	-0.11	(-0.25,0.03)	-0.10	(-0.23,0.04)
South East	-0.11	(-0.24,0.03)	-0.08	(-0.21,0.05)	-0.13	(-0.26,-0.01)	-0.12	(-0.24,0.00)
South West	-0.29	(-0.44,-0.13)	-0.24	(-0.39,-0.08)	-0.27	(-0.41,-0.12)	-0.27	(-0.41,-0.13)
**Overall CAMI**								
North East	Reference		Reference		Reference		Reference^e^	
North West	-0.20	(-0.33,-0.06)	-0.12	(-0.25,0.00)	-0.13	(-0.24,-0.02)	-0.12	(-0.23,-0.01)
Yorkshire and Humber	-0.05	(-0.18,0.07)	-0.04	(-0.16,0.09)	-0.06	(-0.17,0.06)	-0.04	(-0.15,0.08)
East Midlands	-0.12	(-0.24,0.01)	-0.09	(-0.22,0.04)	-0.11	(-0.23,0.01)	-0.08	(-0.20,0.03)
West Midlands	-0.08	(-0.23,0.06)	-0.06	(-0.20,0.08)	-0.08	(-0.20,0.05)	-0.07	(-0.19,0.05)
East of England	-0.04	(-0.15,0.07)	-0.02	(-0.12,0.08)	-0.04	(-0.13,0.06)	-0.02	(-0.12,0.07)
London	-0.21	(-0.35,-0.07)	-0.05	(-0.18,0.08)	-0.13	(-0.26-,0.01)	-0.11	(-0.23,0.01)
South East	-0.11	(-0.23,0.00)	-0.09	(-0.20,0.02)	-0.14	(-0.24,-0.04)	-0.13	(-0.23,-0.03)
South West	-0.25	(-0.39,-0.12)	-0.22	(-0.36,-0.09)	-0.25	(-0.38,-0.12)	-0.25	(-0.38,-0.12)

a. Age was included as a dummy categorical variable in categories shown in Table 1, as the association with overall CAMI departed from linearity.

^b^. Income was included as a continuous variable

^c^. P value for region = 0.0369

^d^. P value for region = 0.0175

^e^. P value for region = 0.0034

**Table 4:** All estimates(beta coefficients) from final models(n = 4227).

	Tolerance and support		Prejudice and exclusion		Overall CAMI	
**Region**						
North East	Reference		Reference		Reference	
North West	-0.11	(-0.24,0.02)	-0.13	(-0.27,0.01)	-0.12	(-0.23,-0.01)
Yorkshire and Humber	-0.04	(-0.18,0.09)	-0.03	(-0.17,0.10)	-0.04	(-0.15,0.08)
East Midlands	-0.09	(-0.22,0.05)	-0.08	(-0.21,0.05)	-0.08	(-0.20,0.03)
West Midlands	-0.05	(-0.20,0.10)	-0.08	(-0.22,0.06)	-0.07	(-0.19,0.05)
East of England	-0.02	(-0.13,0.09)	-0.03	(-0.14,0.08)	-0.02	(-0.12,0.07)
London	-0.13	(-0.29,0.03)	-0.10	(-0.23,0.04)	-0.11	(-0.23,0.01)
South East	-0.14	(-0.26,-0.02)	-0.12	(-0.24,0.00)	-0.13	(-0.23,-0.03)
South West	-0.23	(-0.37,-0.09)	-0.27	(-0.41,-0.13)	-0.25	(-0.38,-0.12)
**Gender**						
Male						
Female	0.15	(0.08,0.22)	0.27	(0.21,0.33)	0.21	(0.15,0.26)
**Age**						
16–24	0.00		0.00		0.00	
25–34	0.14	(-0.02,0.30)	-0.06	(-0.20,0.07)	0.04	(-0.08,0.16)
35–44	0.27	(0.12,0.42)	-0.02	(-0.14,0.10)	0.13	(0.01,0.24)
45–54	0.36	(0.21,0.51)	0.15	(0.03,0.27)	0.25	(0.14,0.37)
55–64	0.46	(0.32,0.59)	0.06	(-0.06,0.19)	0.26	(0.15,0.37)
65–74	0.48	(0.34,0.62)	-0.07	(-0.21,0.07)	0.20	(0.09,0.31)
75+	0.59	(0.43,0.76)	-0.26	(-0.41,-0.11)	0.17	(0.04,0.29)
**Ethnicity**						
White	0.00		0.00		0.00	
Black	-0.23	(-0.56,0.11)	-0.54	(-0.82,-0.26)	-0.38	(-0.65,-0.12)
Asian	-0.36	(-0.60,-0.13)	-0.71	(-0.93,-0.49)	-0.54	(-0.69,-0.38)
Mixed	-0.20	(-0.50,0.11)	0.05	(-0.33,0.43)	-0.07	(-0.40,0.25)
Other	-0.35	(-0.86,0.16)	-0.54	(-0.91,-0.17)	-0.44	(-0.84,-0.05)
**Education**						
Degree	0.00		0.00		0.00	
Below degree	-0.21	(-0.29,-0.13)	-0.24	(-0.31,-0.16)	-0.22	(-0.29,-0.16)
No qualification	-0.45	(-0.58,-0.31)	-0.54	(-0.65,-0.42)	-0.49	(-0.59,-0.40)
**Income**^a^	0.01	(-0.01,0.02)	0.01	(0.00,0.02)	0.01	(0.00,0.02)
**Familiarity with mental illness**						
Yourself	0.00		0.00		0.00	
Other	-0.08	(-0.22,0.07)	-0.06	(-0.21,0.10)	-0.07	(-0.190.06)
Don’t known anyone with mental illness	-0.40	(-0.55,-0.25)	-0.46	(-0.62,-0.30)	-0.43	(-0.56–0.30)
**P-value for region**^b^	*0*.*0369*		*0*.*0175*		*0*.*0034*	

^a^. Income estimate reflects change in attitudes accompanied by an increase in annual salary of £10,000.

^b^. P values for the association between mental-health related attitudes and region are derived from Wald tests, and italicised where they fall below p = 0.05

**Table 5:** Description of study population in terms of mental health-related attitudes, based on responses to the CAMI, from the analysed sample (N = 4277).

	Overall frequency (weighted %)	Tolerance and support		Prejudice and exclusion		Overall CAMI	
		Mean	(95%CI)	Mean	(95%CI)	Mean	(95%CI)
**Region**							
North East	408(4.69)	0.12	(0.02,0.23)	0.17	(0.07,0.27)	0.15	(0.06,0.24)
North West	472(12.22)	-0.04	(-0.16,0.08)	-0.06	(-0.19,0.07)	-0.05	(-0.15,0.05)
Yorkshire and Humber	419(11.07)	0.06	(-0.05,0.18)	0.12	(0.01,0.23)	0.09	(0.00,0.18)
East Midlands	378(8.23)	0.02	(-0.08,0.12)	0.04	(-0.07,0.16)	0.03	(-0.06,0.12)
West Midlands	377(9.72)	0.07	(-0.06,0.20)	0.06	(-0.08,0.19)	0.06	(-0.05,0.18)
East of England	565(11.99)	0.09	(0.02,0.16)	0.12	(0.04,0.21)	0.11	(0.04,0.17)
London	381(14.41)	-0.10	(-0.23,0.02)	-0.02	(-0.16,0.11)	-0.06	(-0.18,0.05)
South East	717(17.03)	0.00	(-0.08,0.09)	0.06	(-0.03,0.15)	0.03	(-0.04,0.11)
South West	510(10.63)	-0.09	(-0.20,0.01)	-0.12	(-0.24,0.00)	-0.11	(-0.21,0.00)
**Age**							
16–24	313(13.32)	-0.32	(-0.45,-0.20)	0.01	(-0.10,0.13)	-0.16	(-0.26,-0.06)
25–34	581(17.54)	-0.10	(-0.19,0.00)	0.05	(-0.06,0.16)	-0.03	(-0.11,,0.06)
35–44	736(17.42)	0.02	(-0.07,0.11)	0.07	(-0.01,0.15)	0.04	(-0.03,0.12)
45–54	805(17.8)	0.09	(0.01,0.17)	0.23	(0.16,0.31)	0.16	(0.09,0.23)
55–64	690(14.51)	0.16	(0.08,0.23)	0.11	(0.03,0.19)	0.13	(0.06,0.20)
65–74	711(11.79)	0.11	(0.03,0.19)	-0.11	(-0.21,-0.01)	0.00	(-0.08,0.07)
75+	391(7.62)	0.11	(0.01,0.21)	-0.45	(-0.55,-0.34)	-0.17	(-0.25,-0.08)
**Gender**							
Male	1885(48.93)	-0.09	(-0.14,-0.03)	-0.12	(-0.17,-0.06)	-0.10	(-0.15,-0.06)
Female	2342(51.07)	0.09	(0.05,0.13)	0.17	(0.13,0.22)	0.13	(0.09,0.17)
**Education**							
Degree	1189(29.69)	0.17	(0.10,0.24)	0.27	(0.20,0.34)	0.22	(0.16,0.28)
Below degree	2261(54.23)	-0.03	(-0.08,0.01)	0.04	(-0.01,0.08)	0.00	(-0.04,0.04)
None	777(16.08)	-0.19	(-0.29,-0.08)	-0.42	(-0.51,-0.33)	-0.30	(-0.37,-0.23)
**Ethnicity**							
White	3899(90.33)	0.05	(0.01,0.08)	0.10	(0.06,0.13)	0.07	(0.04,0.10)
Black	79(2.08)	-0.29	(-0.61,0.03)	-0.52	(-0.81,-0.23)	-0.40	(-0.67,-0.14)
Asian	180(5.36)	-0.49	(-0.74,-0.25)	-0.73	(-0.92,-0.54)	-0.61	(-0.75,-0.47)
Mixed	43(1.40)	-0.37	(-0.63,-0.10)	0.01	(-0.32,0.33)	-0.18	(-0.46,0.10)
Other	26(0.84)	-0.36	(-0.86,0.13)	-0.43	(-0.79,-0.06)	-0.39	(-0.80,0.01)
**Income**							
0.00–232.99	669(16.92)	-0.12	(-0.22,-0.01)	-0.15	(-0.25,-0.05)	-0.13	(-0.21,-0.05)
233.00–368.99	628(14.41)	-0.11	(-0.21,-0.01)	-0.16	(-0.25,-0.08)	-0.14	(-0.22,-0.06)
369.00–531.99	965(21.90)	-0.03	(-0.11,0.05)	-0.04	(-0.12,0.04)	-0.03	(-0.10,0.04)
532.00–851.99	998(23.82)	0.05	(-0.03,0.12)	0.16	(0.08,0.24)	0.10	(0.04,0.17)
852.00 and above	967(22.95)	0.14	(0.07,0.21)	0.23	(0.16,0.29)	0.18	(0.13,0.24)
**Neighbourhood deprivation**							
Least	994(22.32)	0.10	(0.04,0.17)	0.17	(0.09,0.24)	0.14	(0.07,0.20)
	897(20.63)	0.01	(-0.06,0.09)	0.04	(-0.03,0.12)	0.03	(-0.04,0.10)
	848(20.09)	0.06	(-0.02,0.13)	0.08	(0.00,0.16)	0.07	(0.01,0.13)
	789(20.06)	-0.07	(-0.17,0.02)	-0.05	(-0.16,0.05)	-0.06	(-0.15,0.02)
Most	699(16.90)	-0.12	(-0.23,-0.02)	-0.11	(-0.22,0.00)	-0.12	(-0.20,-0.03)
**Urban-rural**							
City	3346(81.08)	-0.02	(-0.06,0.02)	0.02	(-0.03,0.06)	0.00	(-0.04,0.04)
Town or fringe	441(9.31)	0.04	(-0.05,0.13)	0.06	(-0.05,0.16)	0.05	(-0.04,0.13)
Village or hamlet	440(9.61)	0.15	(0.06,0.24)	0.15	(0.04,0.25)	0.15	(0.06,0.23)
**Familiarity with mental illness**							
Yourself	191(3.93)	0.19	(0.05,0.33)	0.22	(0.06,0.38)	0.20	(0.07,0.34)
Other	2748(65.85)	0.12	(0.08,0.16)	0.21	(0.17,0.26)	0.17	(0.13,0.20)
Don’t know anyone with mental illness	1288(30.22)	-0.28	(-0.35,-0.20)	-0.38	(-0.45,-0.31)	-0.33	(-0.39,-0.27)

### Ethics statement

Ethical approval for HSE 2014 was obtained from the Oxford A Research Ethics Committee (12/SC/0317). Data were analyzed anonymously and no consent for this analysis was sought. The authors assert that all procedures contributing to this work comply with the ethical standards of the relevant national and institutional committees on human experimentation and with the Helsinki Declaration of 1975, as revised in 2008.

## Results

Of the overall survey sample of 10,080 participants, data from a total of 7079(70%) respondents were complete for mental health-related attitudes. Of these, 4277 respondents had complete data on the final modelled variables, and formed the final analytic sample. Unadjusted scores showed that London had the most negative mental health-related attitudes on both scales, and overall, with the North East consistently displaying the most positive attitudes (Table 1). Attitudes were more negative in males, in those with fewer educational qualifications, and among the youngest age groups compared to the oldest age groups. For age, tolerance scores became more positive with increasing age, whereas prejudice and exclusion scores increased to a peak at the 45–54 age group, before falling with increasing age. This was also reflected in the relationship between age and overall stigma. Increasing educational attainment was accompanied by generally more positive attitudes, including those on overall stigma. Attitudes on prejudice and exclusion, and overall stigma, were most positive among white people, then mixed ethnicity, then black respondents, then Asian people, and then the “other” ethnic group. Trends for tolerance and exclusion were similar, except that Asian respondents reported the most negative attitudes on this dimension. Attitudes were more positive with increasing income, and with decreasing neighbourhood deprivation. Compared to respondents living in rural settings, respondents in cities were more likely to endorse negative attitudes, both on the overall score and the two sub-domains. Finally, increasing familiarity with mental illness was associated with more positive scores on the overall mental health-related attitudes variable and both factors.

Table 2 shows unadjusted and age- and gender-adjusted association of covariates with mental health-related attitudes. Positive scores on the overall CAMI, and both factors, were associated with female gender, living in a less built up area, lower neighbourhood deprivation, higher educational attainment, white ethnic group, increasing income, and increasing familiarity with mental illness. There were inconsistent trends in associations with age groups between the two dimensions.

See Table 3 for model estimates for region differences in mental health-related attitudes. North East respondents had on average the most positive mental health attitudes, for the overall measure and both dimensions of stigma, after adjustments. For tolerance and support, London respondents reported significantly more negative attitudes compared to the North East, which was attenuated by adjusting for individual characteristics, which included age, gender, ethnic group, income, and educational attainment. More negative attitudes on this dimension in residents of the South East and South West became more pronounced on adjustment, and were statistically significant in final models. There was statistical evidence for overall association between region and tolerance and support, after all adjustments(p = 0.0369). Compared to survey participants living in the North East, statistically more negative attitudes for prejudice and exclusion were noted in those living in the North West, London and the South West in unadjusted models. Adjustment for covariates attenuated estimated negative differences between the North East reference group and London, and the North West, while negative differences between the North East and the South West were made more pronounced. In fully adjusted models, respondents from the South East and South West reported more negative scores on prejudice and exclusion compared to the North East. Regional associations were similar for the combined mental health stigma variable, with more negative attitudes in the North West, London, South East, South West of England compared to the North East. Statistical evidence for an overall association between region and overall mental health-related attitudes was found after all adjustments (p = 0.0034).

In fully adjusted models (Table 4), more positive attitudes on tolerance and support were associated with increasing age, female gender, higher educational attainment, white ethnic group, higher income, and greater familiarity. These associations were similar for prejudice and exclusion, however age relationships were more inconsistent- respondents aged 25–34, and 35–44, had slightly lower scores after adjustments, with the 45–54 year olds displaying the most positive scores on this variable, with reducing scores in the older two age groups.

Trends in scores were similar in both the complete data and the data restricted to records with complete data on modelled variables (see Table 5).

## Discussion

### Summary of findings

Statistical differences between English regions were noted for tolerance and support, prejudice and exclusion, and overall mental health-related attitudes. Compared to the North East, more negative attitudes reported by respondents living in the South West, and the South East, remained statistically significant even after accounting for sociodemographic differences in respondents between the regions. London displayed more negative attitudes for both tolerance and support and for prejudice and exclusion, but the magnitude of these differences was attributable partly to individual characteristics of respondents varying between regions. In fully-adjusted models, region remained associated with both dimensions of mental health–related attitudes and overall mental health related attitudes.

### Limitations and strengths

To our knowledge, we present the first assessment of regional variation in mental health-related attitudes in nationally representative data. Our sample was relatively large, and we had access to a broad range of individual level characteristics that could have explained differences in mental health-related attitudes between respondents. Stigma was measured using an accepted mental health attitudes scale that has been used in previous research[20], and allowed the assessment of two aspects of mental health-related attitudes. We took account of individual familiarity with mental illness (although this was limited by missing data), and of a range of possible confounders. Given the study was nationally representative and made use of reasonably well-researched scales, we suggest that our results are generalizable to mental health-related attitudes in the population of England.

On the other hand, only limited geographic resolution was available with which to model regional differences- owing to small number of geographic units, we were unable to carry out multilevel analysis, and unable to fully account for other geographic characteristics which might influence stigma, such as public disorder or prevalence of mental illness. Data was cross-sectional, and from one time-point. Bias from non-response was minimised by the use of survey weights. However it is also possible that non-response to items on the mental illness attitudes scale may also have differed depending on mental health-related attitudes. For example, information on mental health-related attitudes were derived from face-to-face interview and therefore more likely to be affected by desirability bias or agreement bias[25]. We only had data on individual mental health-related attitudes from a national survey sample. However regional differences in mental health stigma could also be reflected in structural differences, for example in funding for mental health services, and local variation in mental health-related discrimination—our study only focused on regional differences in individual mental health-related attitudes. Information on familiarity with mental illness was available on a minority of participants, limiting sample size for multivariable models.

### Explanations

Regional differences in mental health stigma could reflect variation in structural characteristics of areas, that is, aspects of local institutions, organizations, and policies, which may act to label, exclude, and discriminate against people with mental illness[26]. For example, ecological or contextual mental health stigma could be reflected in greater disparities in funding between mental and physical health, or in more discriminatory practices on mental health and the workplace, and less stringent implementation of national guidance and legislation protecting rights of people with mental illness[27]. Media coverage of mental illness also varies by region, but the relationship with media may be more complex- attitudes may be shaped not only by events and their coverage, but also local patterns of engaging with news media[14].

Regional differences in mental health stigma could reflect differing levels of social cohesion. For example more socially cohesive areas, where residents feel a greater trust in other residents and levels of social participation are greater, could also contain individuals with greater familiarity with people with mental illness, resulting in more positive attitudes because of greater contact, in line with work indicating that greater contact with stigmatized groups is associated with more positive attitudes over time[28]. Although we adjusted for familiarity in our analysis, mental health-related attitudes might be affected by more distant contact with people with mental illness, which we were not able to measure, and which might be higher in more cohesive communities. Regions with more cohesive communities might also contain individual with greater familiarity, greater levels of social contact, and less social distance with regards to people with mental illness, resulting in more positive attitudes in regions enclosing more cohesive areas. This is consistent with an “intergroup contact” hypothesis for mental health stigma, in line with evidence on ethnicity[29], sexual orientation, and physical disability[30], where greater contact with minority groups is accompanied by more positive attitudes towards these groups.

### Previous literature

We extended previous crude descriptions of stigma scores between regions[17], to examine whether regional differences in mental health stigma were explained by individual characteristics. There is limited previous literature examining area influences on mental health stigma. From 2000 to 2003, England showed a greater negative trend in mental health-related attitudes compared to Scotland, based on analysis of the United Kingdom Department of Health Attitude to Mental Illness Surveys[31]. Hansson et al[5] evaluated a stigma campaign delivered in three Swedish regions, finding overall improvements in these regions over time, and subsequently in Sweden as whole. However baseline comparisons between regions in attitudes are not reported, and inter-regional differences in improvement are not displayed, limiting comparison with our results. Using data on self-stigma and public attitudes from 14 European countries, Evans-Lacko and others[32] found that countries with more positive public attitudes towards mental illness were associated with lower levels of self-stigma—however, this study was unable to assess variation in public attitudes within countries, as reported in this paper. A study of 11 postcode areas in New York City[33] found that area-level mental health-related attitudes did not predict stigma experienced by people with mental health problems. Min and Wong[34] examined relations between community-level factors and the experience of mental illness stigma in South Korea, measuring neighbourhood-level factors at the level of areal units of 20000 residents, in 532 individuals diagnosed with serious mental illness. In this study, the proportion of disabled people in each neighbourhood was positively associated with reporting greater experienced mental health stigma, but did not measure mental illness stigma in the population as a whole. Gaddis et al[35] examined school-level stigma in relation to a range of mental illnesses in American higher education data, finding only weak statistical evidence (p<0.1) for association between higher school-level stigma and individual suicidal ideation and self-injury. Differences in stigma between schools is reported as a range, and neither statistical differences in school-level stigma, nor explanations for any differences, were directly examined. It is generally considered that more negative attitudes towards mental illness exist in more rural areas[36, 37] compared to urban areas, although there have been few direct comparisons of attitudes between rural and urban residents[38]. Liu et al[9] report more positive mental health-related attitudes among residents of three rural districts of Beijing(Haidian, Daxing, and Pinggu), compared to the more urban reference group (Xuanwu), after adjusting for age, educational level, income, self-rated health, employment status, and the presence of health insurance. In a longitudinal study of American adults, Hoyt et al(1997) reported greater depressive symptomatology, greater mental health stigma, and reduced willingness to seek help in participants residing in towns with smaller populations. Overall, our results are consistent with models of stigma intervention that adopt a multilevel approach, insofar as these models conceptualize stigma as a product of personal, inter-personal, and structural influences[39].

## Conclusions

We found that region of residence remained associated with tolerant attitudes towards people with mental illness, and mental health-related prejudice, as well as overall mental health-related attitudes, despite adjustments for individual characteristics including familiarity with mental illness, age, ethnicity, education, and income. Future research should focus on testing whether contextual effects of mental health-related attitudes exist, in multilevel analyses with sufficient geographic detail. Such relationships could be relevant to the future design and implementation of stigma interventions.
